# Iohexol clearance is superior to creatinine-based renal function estimating equations in detecting short-term renal function decline in chronic heart failure

**DOI:** 10.3325/cmj.2015.56.531

**Published:** 2015-12

**Authors:** Katja Cvan Trobec, Mojca Kerec Kos, Stephan von Haehling, Stefan D. Anker, Iain C. Macdougall, Piotr Ponikowski, Mitja Lainscak

**Affiliations:** 1 Faculty of Pharmacy, University of Ljubljana, Ljubljana, Slovenia; 2 Innovative Clinical Trials, Department of Cardiology & Pneumology, University Medical Center Göttingen (UMG), Göttingen, Germany; 3 Department of Renal Medicine, King's College Hospital, Denmark Hill, London, UK; 4 Medical University, Wroclaw, Poland; 5 Department of Cardiology and Department of Research and Education, General Hospital Celje, Celje, Slovenia; 6 Faculty of Medicine, University of Ljubljana, Ljubljana, Slovenia

## Abstract

**Aim:**

To compare the performance of iohexol plasma clearance and creatinine-based renal function estimating equations in monitoring longitudinal renal function changes in chronic heart failure (CHF) patients, and to assess the effects of body composition on the equation performance.

**Methods:**

Iohexol plasma clearance was measured in 43 CHF patients at baseline and after at least 6 months. Simultaneously, renal function was estimated with five creatinine-based equations (four- and six-variable Modification of Diet in Renal Disease, Cockcroft-Gault, Cockcroft-Gault adjusted for lean body mass, Chronic Kidney Disease Epidemiology Collaboration equation) and body composition was assessed using bioimpedance and dual-energy x-ray absorptiometry.

**Results:**

Over a median follow-up of 7.5 months (range 6-17 months), iohexol clearance significantly declined (52.8 vs 44.4 mL/[min ×1.73 m^2^], *P* = 0.001). This decline was significantly higher in patients receiving mineralocorticoid receptor antagonists at baseline (mean decline -22% of baseline value vs -3%, *P* = 0.037). Mean serum creatinine concentration did not change significantly during follow-up and no creatinine-based renal function estimating equation was able to detect the significant longitudinal decline of renal function determined by iohexol clearance. After accounting for body composition, the accuracy of the equations improved, but not their ability to detect renal function decline.

**Conclusions:**

Renal function measured with iohexol plasma clearance showed relevant decline in CHF patients, particularly in those treated with mineralocorticoid receptor antagonists. None of the equations for renal function estimation was able to detect these changes.

**ClinicalTrials.gov registration number:**

NCT01829880

Renal function is one of the key clinical parameters to be monitored in heart failure patients; it predicts mortality and can be crucial for making therapeutic decisions (1,2). The “gold standard” for renal function assessment is the measurement of inulin clearance, which is difficult to perform and not feasible in routine work (3). An alternative approach is measurement of iohexol clearance, which correlates well with inulin clearance and is considered a robust standard for evaluating renal function (4,5). In clinical practice, renal function is also estimated by equations based on serum creatinine concentration, for example, the Cockcroft-Gault equation (CG), four- and six-variable Modification of Diet in Renal Disease equation (MDRD4 and MDRD6), and the more recently described Chronic Kidney Disease Epidemiology Collaboration equation (CKD-EPI) (6-8). Recently, the CKD-EPI equation was shown to outperform the MDRD4 equation in chronic heart failure (CHF) patients in terms of accuracy and precision (9) and for mortality prediction (10,11).

Accuracy of equations based on serum creatinine concentration is affected by different demographic variables. Serum creatinine concentration depends on renal function but is also associated with muscle mass, ie, body composition (12). Generally, inclusion of lean body mass into the calculation improved the glomerular filtration rate (GFR) estimation (13). However, some studies suggest that this is restricted to overweight patients (14). These studies were performed in patients with chronic kidney disease or healthy individuals, and there is a lack of information on CHF patients.

A significant proportion of CHF patients with systolic dysfunction develop a rapid decline in renal function, regardless of their baseline renal function. Among all patients with heart failure, the prevalence of some degree of chronic kidney disease is between 25% and 63% (15,16). In this population, the rate of decline is a strong predictor of increased mortality (1). Previous studies that investigated the performance of different equations over time were mostly performed in patients with kidney disease and no similar study was conducted in patients with CHF (17,18).

We aimed to compare the performance of iohexol plasma clearance and creatinine-based glomerular filtration estimating equations (CG, MDRD, CKD-EPI) in 6-month renal function estimation in CHF patients. We also investigated the association of estimates with body composition evaluated using bioimpedance analysis (BIA) and dual-energy x-ray absorptiometry (DEXA).

## Materials and methods

### Participants and study design

Consecutive patients with CHF diagnosed according to European Society of Cardiology guidelines attending our outpatient clinic were screened for inclusion between November 2011 and February 2013. We excluded patients who had estimated glomerular filtration rate (eGFR) with MDRD4 < 30 mL/ (min ×1.73 m^2^) at baseline visit to maintain iohexol sampling interval of 4 hours (5,19). Measurements and patient evaluation were performed at baseline and at least 6 months thereafter (follow-up). All patients had to be without acute deterioration of heart failure (requiring hospitalization or change in therapy) for at least four weeks before both visits. Patients attended the clinic in the morning, after overnight fasting, and before taking their prescribed drug therapy. Blood biomarkers, iohexol clearance, and body composition were determined at both visits. The protocol was approved by the Republic of Slovenia National Medical Ethics Committee and the trial was registered at ClinicalTrials.gov (NCT01829880). Patients received verbal and written information about the study and provided written informed consent before any study-related procedure.

### Determination of measured glomerular filtration rate (mGFR)

Iohexol solution (5 mL of Omnipaque 300 ®, GE Healthcare, Cork, Ireland) was administered by intravenous injection. Blood samples were drawn into lithium heparinized tubes before administration of iohexol 3 hours and 4 hours post administration. Blood was centrifuged immediately (15 min, 5°C, 3500 g) and plasma was stored at -80°C for further analyses. The samples were analyzed on a continuous basis (to avoid any storage driven analytical mistakes) throughout the study. Plasma concentration of iohexol was measured as described later in the text. Clearance of iohexol was calculated based on a one-compartment model utilizing measured 3-hour and 4-hour iohexol concentrations and applying Bröchner-Mortensen correction (4,20).

### Blood biomarkers

Blood biomarkers (serum creatinine, urea, albumin, N-terminal pro B-type natriuretic peptide, electrolytes, and complete blood count) were determined in a blood sample drawn before administration of iohexol. Serum creatinine concentration was measured by the kinetic Jaffe reaction, rate-blanked and compensated on a Cobas 6000 analyzer (Roche Diagnostics, Basel, Switzerland). The method is standardized to Isotope Dilution Mass Spectrometry method.

### Determination of estimated glomerular filtration rate (eGFR)

eGFR was calculated on the basis of serum creatinine concentration and other variables using the following five equations: CG, CG adjusted for lean body mass (GCLBM), MDRD4, MDRD6, and CKD-EPI equation (6-8). Creatinine clearance (ClCr) using CGLBM was calculated as shown in Equation 1. Lean body mass (LBM), used for calculation, was measured with DEXA or (in patients where DEXA was not performed) with BIA.


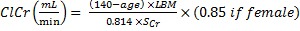
 Equation 1

S_Cr_ is serum creatinine concentration (in μmol/L). eGFR was calculated in mL/ (min ×1.73 m^2^) for all five equations. To transform the units of CG and CGLBM equations from mL/min to mL/(min ×1.73 m^2^), body surface area was calculated using the DuBois equation (21).

### Body composition measurement

At both visits, body composition was determined by BIA and DEXA. BIA was performed in fasting patients after 10 minutes of supine rest with Bodystat 1500 analyzer (Bodystat Ltd, Isle of Man, UK). Fat mass (in kg and %), lean mass (in kg and %), dry lean mass (in kg and %), and water content (in liters and %) was recorded. Four patients with pacemakers did not undergo this test.

Full body scan DEXA was performed with Hologic Explorer, QDR Series (Hologic Inc., Waltham, MA, USA) to assess fat mass (in kg and %), lean mass (in kg and %), and bone mineral content (in kg). The sum of lean mass in both arms and legs was calculated to obtain appendicular skeletal muscle mass, and skeletal muscle index was calculated with Equation 2 (22). Fat-free mass was calculated as the sum of lean mass and bone mineral content measured by DEXA, and fat-free mass index was calculated using Equation 3. A DEXA scan was not recorded in one patient at baseline and in three patients at follow-up due to lack of availability of the device at the time of the visit.


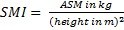
 Equation 2

Where SMI is skeletal muscle index and ASM is appendicular skeletal muscle mass.


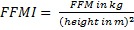
 Equation 3

Where FFMI is fat-free mass index and FFM is fat-free mass.

### Measurement of iohexol in plasma

Plasma concentration of iohexol was measured with an isocratic reversed-phase high-performance liquid chromatography coupled with an UV detector. The method, which was previously described (23), was internally optimized and validated before use. 200 μL of 5% perchloric acid was added to 100 μL of plasma and mixed with a vortex mixer for 3 min. The samples were put in an ultrasonic bath for 5 min, followed by centrifugation (10 min, 10 000 g, 4°C). 70 μL of 2.5% ammonia solution was added to 100 μL of the supernatant and mixed with a vortex mixer for 30 s. The obtained solution was analyzed by high-performance liquid chromatography system. The chromatographic separation was achieved on a Synergi Hydro 250 × 4.60 mm column with 4 µm particles, guarded by a C18 guard column (Phenomenex, Torrance, CA, USA) at 40°C. The mobile phase consisted of a mixture of 50 mM phosphate buffer (pH 3): acetonitrile (94:6%v/v) and the flow rate was 1.0 mL/min. The UV-detection was carried out at 254 nm.

### Statistical analysis

Data are presented with a mean value ± standard deviation or median and range. Two tailed *P* values lower than 0.05 were considered statistically significant. Kolmogorov-Smirnov test showed a normal distribution of all values included in linear regression and/or paired sample *t* tests (*P* > 0.05). All calculations were performed with SPSS for Windows 19.0 (IBM Corp, Armonk, NY, USA).

Linear regression was used to determine the extent of linear dependence between numerical patients' characteristics and mGFR and changes in mGFR (Pearson correlation). Paired sample *t* test was used to compare mGFR and patients' parameters at both visits, and an independent sample *t* test was used to compare the influence of different categorical variables on mGFR. In cases of linear regression with multiple comparisons employed on the same data set, Bonferroni correction was used. The paired sample *t* test with multiple comparisons was done in SPSS and *P* values were adjusted for Bonferroni correction.

Absolute differences between eGFR and mGFR were calculated with Equation 4 and PE with Equation 5.


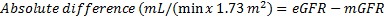
 Equation 4


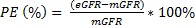
 Equation 5

Where eGFR is estimated glomerular filtration rate, mGFR is measured glomerular filtration rate, and PE is percentage error.

Mean PE, absolute difference in mL/(min ×1.73 m^2^), percentage of estimated GFR within 30% of measured GFR (P30), and correlation coefficients were calculated for all equations at both visits. Bland-Altman plots were drawn for all equations at baseline. Linear regression was used to evaluate PE of equations with both mGFR and parameters of body composition at baseline. Additionally, multiple linear regression was performed to assess whether equations with included percentage lean body mass (measured with DEXA at baseline visit) better predicted mGFR (α = 0.001, “enter” method). Percentage lean body mass was chosen to be included into the model on the basis of results of Pearson correlation between various body composition parameters and mGFR.

## Results

**Figure 1:**
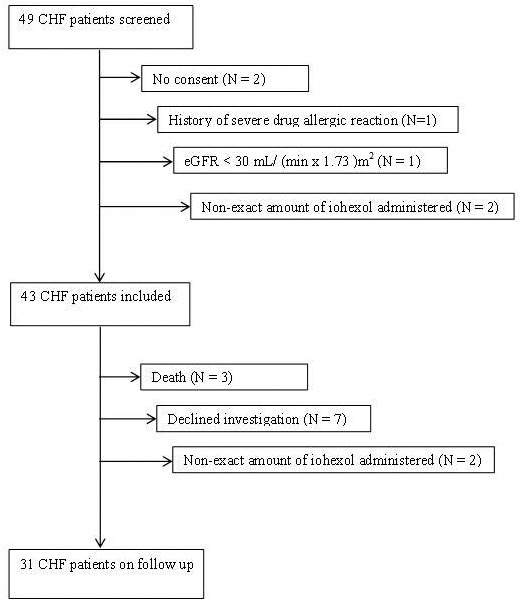
Flowchart.

49 patients were screened for inclusion. 2 patients did not consent to take part in the investigation, 1 patient reported a history of severe allergic reactions to drugs, 1 patient had an eGFR below 30 mL/(min ×1.73 m^2^), and 2 patients were additionally excluded from the analysis due to inappropriate iohexol administration. Accordingly, 43 patients with CHF (58% male, mean age 73 years) were included into the study, and 31 patients had a follow-up iohexol assessment (3 patients died, 7 declined to participate, and 2 had inappropriate iohexol administration) (Figure 1). The mean follow-up time was 8 months (median 7.5 months, range 6-17 months). Baseline patients’ characteristics and renal function are presented in Table 1. 16 patients (37%) had left ventricular ejection fraction ≤40%. Their mean mGFR was 53.1 mL/(min ×1.73 m^2^), with mGFR below 30 mL/(min ×1.73 m^2^) in 2 (5%) patients, between 30 and 60 mL/(min ×1.73 m^2^) in 26 (61%) patients, and over 60 mL/(min ×1.73 m^2^) in 15 (35%) patients.

**Table 1:** Baseline patients’ characteristics (N = 43)*

Characteristic	
Sex (% male)	58
NYHA class I/II/III, N (%)	2 (5)/35 (81) /6 (14)
	Mean (standard deviation)
Age (years)	73 (9)
Weight (kg)	81 (18)
BMI	29.5 (6.0)
BSA (m^2^)	1.9 (0.2)
Mean SBP (mmHg)	131 (18)
Mean DBP(mmHg)	71 (11)
Mean heart rate (min^-1^)	72 (16)
LVEF (%)	47.8 (13.3)
Hematocrit (%)	40.0 (4.0)
Serum NT-proBNP (ng/L)	2329 (2,161)
Serum urea (mmol/L)	9.6 (3.6)
Serum albumin (g/L)	44.9 (2.9)
Serum creatinine (μmol/L)	105 (28)
CL iohexol (mL/[min ×1.73 m^2^])	53.1 (17.7)
	Percent
**Concomitant diseases**	
Hypertension	72
Atrial fibrillation	58
Coronary artery disease	21
Diabetes mellitus	16
Chronic obstructive pulmonary disease	5
Hypercholesterolemia	54
**Medication**	
ACEi or ARB	98
Beta blockers	100
MRA	58
Loop diuretics	63
Thiazide or thiazide-like diuretics	19
Warfarin	67
Antiaggregation therapy	23
Methyldigoxin	19
Calcium channel blockers	19
Statins	56

*NYHA – New York Heart Association Functional Classification; BMI – body mass index; BSA – body surface area; SBP – systolic blood pressure; DBP – diastolic blood pressure; NT-proBNP – N-terminal prohormone of brain natriuretic peptide; CL – clearance; ACEi – angiotensin-converting-enzyme inhibitor; ARB – angiotensin receptor blocker; MRA – mineralocorticoid receptor antagonist.

At baseline, mGFR was inversely correlated with age (r = −0.354, *P* = 0.002). Among concomitant diseases and drug therapy, only arterial hypertension was associated with poorer renal function (52.9 vs 69.4 mL/min, *P* = 0.014). Different parameters of body composition were correlated with baseline mGFR: fat mass in kg (r = -0.331, *P* = 0.032), percentage fat mass (r = -0.383, *P* = 0.012), and percentage lean mass (r = 0.388, *P* = 0.011).

**Figure 2:**
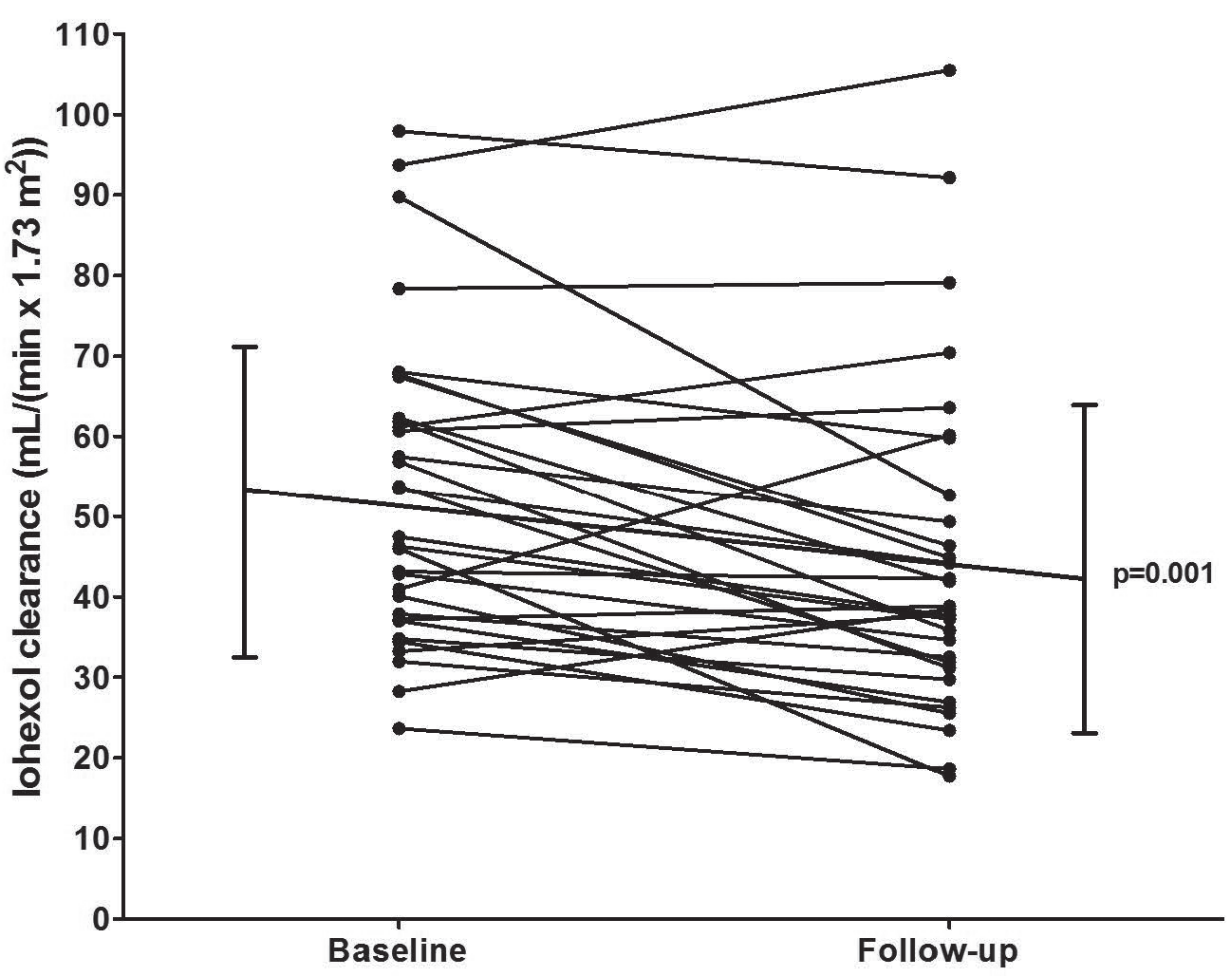
Decline in measured glomerular filtration rate during follow up (N = 31).

Serum biomarkers did not significantly change between the two visits, except for serum albumin (Table 2). Body weight and most parameters of body composition (except body fat and lean mass) also remained stable (Table 2). mGFR significantly declined during the follow-up (from 52.8 to 44.4 mL/[min ×1.73 m^2^], *P* = 0.001, Figure 2 and Supplementary Figure 1(web extra figure 1)). Serum creatinine concentration remained unchanged, leaving the decline in mGFR undetected by equations (Table 3). In 6 patients, mGFR decreased from >60 mL/(min ×1.73 m^2^) to 30-60 mL/(min ×1.73 m^2^) and in additional 6 patients from 30-60 mL/(min ×1.73 m^2^) to <30 mL/(min ×1.73 m^2^).

**Table 2:** Measured renal function, serum markers, and body composition parameters at baseline and follow-up (N = 31)*

	Baseline mean (SD)	Follow-up mean (SD)	*P*
CL iohexol (mL/[min ×1.73 m^2^])	52.8 (19.1)	44.4 (20.6)	**0.001**
Serum creatinine (μmol/L)	106 (28)	108 (33)	0.561
Serum urea (mmol/L)	9.8 (3.9)	9.6 (3.7)	0.700
Serum albumin (g/L)^†^	45.1 (3.0)	43.7 (3.5)	**0.014**
Body weight (kg)	83.0 (18.6)	83.0 (19.7)	0.954
BMI	30.3 (5.7)	30.2 (6.2)	0.805
BSA	1.90 (0.24)	1.90 (0.25)	0.968
**BIA (N = 27)**			
Fat (kg)	30.1 (10.3)	31.1 (11.7)	0.092
Fat (%)	36.0 (8.5)	37.0 (9.2)	**0.033**
Lean (kg)	53.3 (13.1)	52.4 (13.5)	0.080
Lean (%)	64.1 (8.4)	63.0 (9.2)	**0.021**
Dry lean (kg)	10.6 (5.1)	10.4 (5.5)	0.197
Dry lean (%)	12.2 (4.7)	11.8 (5.3)	0.106
Water (L)	42.6 (8.2)	42.0 (8.3)	0.104
Water (%)	51.8 (6.4)	51.2 (7.2)	0.275
**DEXA (N = 27)**			
Fat (kg)	27.2 (11.0)	28.0 (10.4)	**0.044**
Fat (%)	32.7 (8.8)	33.4 (7.8)	0.112
Lean (kg)	51.9 (10.8)	52.0 (11.1)	0.753
Lean (%)	64.4 (8.3)	63.6 (7.4)	0.081
SMI	7.27 (1.45)	7.37 (1.41)	0.454
FFMI	19.27 (2.49)	19.31 (2.80)	0.741

*CL – clearance; BMI – body mass index; BSA – body surface area; BIA – bioimpedance analysis; DEXA – dual-energy x-ray absorptiometry; BMC – bone mass content; FFMI – fat-free mass index; SMI – skeletal muscle index; SD – standard deviation.

†N = 29.

**Table 3:** Performance of equations on baseline and follow-up*

	Baseline (N = 43)					Follow-up (N = 31)				
	Mean GFR (mL/[min ×1.73 m^2^])	MPE (%)	Mean absolute difference (mL/[min ×1.73 m^2^])	Pearson r^2 ‡^	P30 (%)	Mean GFR (mL/[min ×1.73 m^2^])	MPE (%)	Mean absolute difference (mL/[min ×1.73 m^2^])	Pearson r^2 §^	P30 (%)
CL iohexol	53.1					44.4				
MDRD4	57.9	11.6	4.83	0.712	88.4	56.2	33.6	11.8	0.759	51.6
MDRD6	61.2^†^	11.7^†^	7.67^†^	0.699	78.6	59.8^‡^	41.3^‡^	15.1^‡^	0.740	43.3
CG	57.9	12.0	4.79	0.537	72.1	57.5	35.7	13.1	0.781	48.4
CGLBM	36.4	- 30.5	- 16.7	0.726	44.2	36.2	- 15.9	- 8.23	0.832	74.2
CKD-EPI	49.3	- 4.86	- 3.75	0.540	81.4	49.2	16.7	4.80	0.658	74.2

*GFR – glomerular filtration rate; MPE – mean percentage error; MDRD4 – four-variable Modification of Diet in Renal Disease equation; MDRD6 – six-variable Modification of Diet in Renal Disease equation; CG – Cockcroft-Gault equation; CGLBM –Cockcroft-Gault equation adjusted for lean body mass; CKD-EPI – Chronic Kidney Disease Epidemiology Collaboration equation. P30 – percentage of estimated GFR within 30% of measured GFR.

†N = 42.

‡N = 30.

§*P* < 0.001 for all equations.

**Figure 3:**
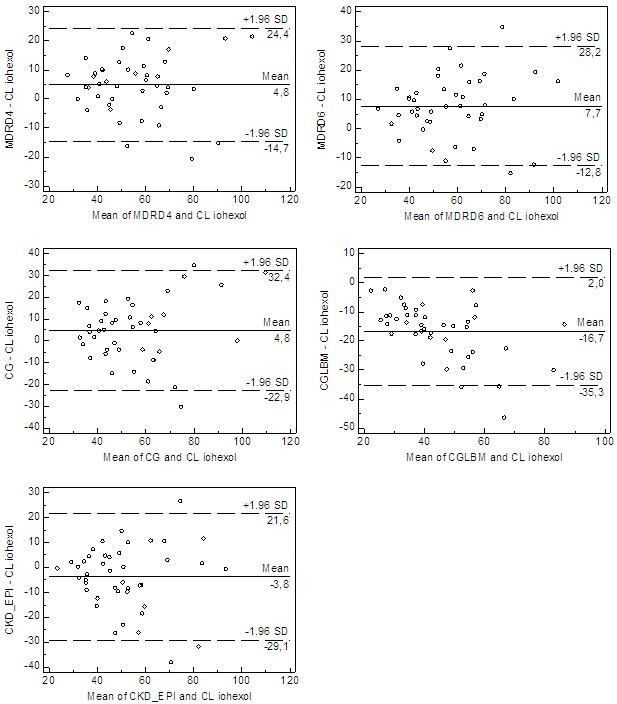
Bland-Altman plots for creatinine-based renal function estimating equations at baseline visit. CL – clearance, CG – Cockcroft-Gault equation, CGLBM –Cockcroft-Gault equation adjusted for lean body mass, CKD-EPI – Chronic Kidney Disease Epidemiology Collaboration equation, MDRD4 – four-variable Modification of Diet in Renal Disease equation, MDRD6 – six-variable Modification of Diet in Renal Disease equation.

The mean PE and absolute differences between eGFR and mGFR tended to increase toward positive values from baseline to follow-up visit (Table 3). CKD-EPI had the lowest mean absolute difference between eGFR and mGFR as well as the mean PE. Also CG, MDRD4, and MDRD6 estimated renal function at baseline considerably well, but at follow-up they overestimated renal function by more than 30%. CGLBM was the only equation that underestimated GFR at both visits. Bland-Altman plots for all equations at baseline are presented in Figure 3. PE of all equations was inversely correlated with mGFR, which suggests that they overestimate renal function when mGFR levels are low and underestimate it when mGFR levels are high (data not shown).

The accuracy of CG, CGLBM, and CKD-EPI equations was associated with body composition parameters, while the accuracy of MDRD4 and MDRD6 was not (Supplementary Table 1 (web extra table 1)and Supplementary Table 2(web extra table 2)). Linear regression showed that mass was positively associated, while lean body mass and water were negatively associated with PE. Multiple linear regression revealed that additional 23% of the variance in mGFR can be explained by adding percentage of lean body mass (measured with DEXA) to CG equation. This percentage was 15.3% for CKD-EPI and 4.0% for the CGLBM equation, while the improvement did not reach statistical significance for MDRD4 and MDRD6 equations (Supplementary Table 2(web extra table 2)).

**Figure 4:**
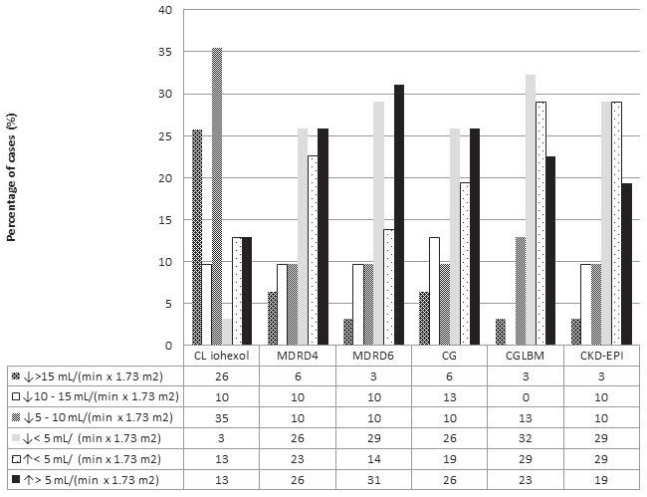
Decline of renal function during follow-up (N = 31). CG – Cockcroft-Gault equation, CGLBM – Cockcroft-Gault equation adjusted for lean body mass, CKD-EPI – Chronic Kidney Disease Epidemiology Collaboration equation, CL – clearance, MDRD4 – four-variable Modification of Diet in Renal Disease equation, MDRD6 – six-variable Modification of Diet in Renal Disease equation.

Between the two visits, mGFR declined for more than 15 mL/(min ×1.73 m^2^) in 26% of patients and did not decline in 26% of patients (Figure 4). eGFR declined for more than 15 mL/(min ×1.73 m^2^) in only 3%-6% of patients and did not decline in 45%-52% of patients.

**Figure 5:**
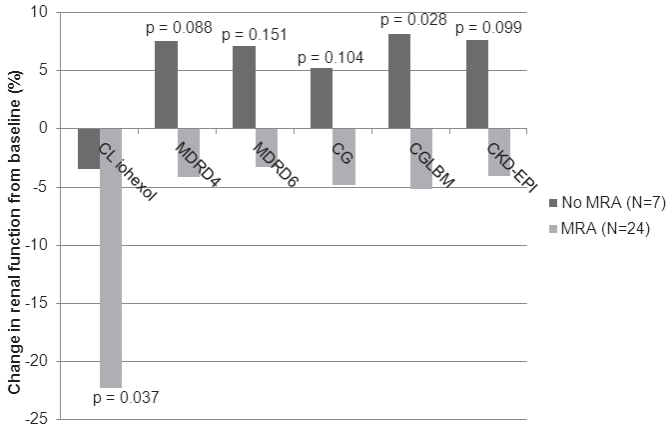
Change in renal function during follow-up according to prescription of mineralocorticoid receptor antagonists at baseline. CG – Cockcroft-Gault equation, CGLBM –Cockcroft-Gault equation adjusted for lean body mass, CKD-EPI – Chronic Kidney Disease Epidemiology Collaboration equation, CL – clearance, MDRD4 – four-variable Modification of Diet in Renal Disease equation, MDRD6 – six-variable Modification of Diet in Renal Disease equation, MRA – mineralocorticoid receptor antagonist (spironolactone or eplerenone).

The decline in mGFR was not related to any baseline characteristic with the exception of mineralocorticoid receptor antagonists (spironolactone or eplerenone) prescription at baseline (Figure 5). Patients receiving mineralocorticoid receptor antagonists at baseline had a mean decline in mGFR of 22% of baseline value and patients not receiving this therapy had a mean decline of 3%. The difference in renal function decline was also detected with equations, although only CGLBM equation detected the difference with statistical significance (*P* < 0.050).

The percentage change in mGFR was correlated with changes in demographic parameters and parameters of body composition between both visits. A significant correlation was observed between change in body weight (r = 0.386, *P* = 0.032), body surface area (r = 0.358, *P* = 0.048), systolic blood pressure (r = 0.405, *P* = 0.024), serum urea concentration (r = −0.507, *P* = 0.004), and serum creatinine concentration (r = −0.427, *P* = 0.016).

## Discussion

This is the first prospective longitudinal study that assessed both measured and estimated renal function in patients with CHF treated according to guidelines. Measurement with iohexol demonstrated a significant decline of renal function, most prominent in patients treated with mineralocorticoid receptor antagonists, those who experienced weight loss, or those with increased serum urea concentration. No equation for estimation of renal function performed similarly to mGFR and thus was unable to detect the significant decline of renal function. In clinical practice, this can cause inappropriate risk stratification or therapeutic decisions with potential harm to the patient.

The decline of renal function in patients with heart failure was previously evaluated by Khan et al (1) using equations for estimation of renal function. In this study, 6640 patients were followed for a mean of 34 months and MDRD4 detected a rapid decline of renal function, with one-third of patients experiencing a decline greater than 5 mL/min per year and an additional third experiencing a decline of up to 5 mL/min per year. However, this was a retrospective analysis, with a potential bias of patients being in a non-stable condition and without validation with an external marker of renal function. In our study, patients were treated according to guidelines and were in a stable condition, the equations detected no decline of renal function in 50% of our patients, while only 25% had stable renal function measured with iohexol. Our results are consistent with other findings regarding the decline in eGFR, but cannot be compared in terms of mGFR since previous studies did not prospectively evaluate mGFR in patients with CHF (1,24).

Mineralocorticoid receptor antagonist usage was the only baseline characteristic that predicted mGFR decline. It is not clear whether this is a consequence of drug therapy alone or of more progressive disease in patients who take mineralocorticoid receptor antagonists. While mineralocorticoid receptor antagonist treatment is beneficial for patients with CHF, it may carry a risk of renal function worsening with increased mortality (25-27).

Renal function was overestimated by both MDRD equations, CG equation at baseline, and CKD-EPI equation at follow-up. As mGFR declined from baseline to follow-up, mean PE and absolute differences tended to increase toward positive values for all equations. Both MDRD equations and CG equation overestimated mGFR by 12 to 15 mL/(min ×1.73 m^2^) at follow-up. Similar absolute overestimation (10 to 15 mL/[min ×1.73 m^2^]) was observed in the study by O’Meara et al (28), where mean mGFR of heart failure patients (46.9 mL/[min ×1.73 m^2^]) was comparable to the mean mGFR in our patients at follow-up (44.6 mL/[min ×1.73 m^2^]). Two other studies in heart failure patients found that equations underestimated mGFR, but these were performed in populations with mean GFR higher than 70 mL/(min ×1.73 m^2^) (9,29). CGLBM equation was the only equation that underestimated mGFR at both visits.

The accuracy of CG, CGLBM, and CKD-EPI equation was related to body composition parameters and could be partly improved by inclusion of lean mass into the calculation. In patients with a lower percentage of lean mass there was a greater risk of renal function overestimation, while in patients with a higher percentage of lean mass there was a greater risk of its underestimation. This could be due to the relationship among lean mass, muscle mass, and serum creatinine concentration. In extremely underweight or obese patients, estimation of renal function with CG and CKD-EPI equations could lead to greater prediction errors, which can be partly improved by body composition measurement.

We were not able to explain equations’ failure to detect renal function decline with changes in body composition. Moreover, MDRD equations, which do not include body composition parameters, also followed the same trend. The reasons for failure are likely to be complex and multifactorial. Previous studies have already shown that equations perform poorly for longitudinal monitoring of renal function because of increased fraction of creatinine tubular secretion in the failing kidney (30,31). Furthermore, the blockade of renin-angiotensin system was shown to further increase tubular secretion of creatinine, leaving serum creatinine concentration unchanged regardless of GFR decline (32). In our study, all patients were treated with angiotensin-converting-enzyme inhibitors or angiotensin receptor blockers. Since this is a fundamental therapy in heart failure, renal function monitoring with equations in this population could be inaccurate.

This study has several limitations. With a study sample of 31 patients who took part in the follow-up, generalizibility of results is limited and confirmatory studies are needed. However, the observed renal function decline was consistent over the entire patient group and was clinically significant. The GFR was estimated at a single point in time at baseline and follow-up and may thus not completely reflect dynamic longitudinal changes of kidney function. Additionally, the MDRD equations were also applied in patients with GFR over 60 mL/(min ×1.73m^2^), although this is not a recommended approach. However, we aimed to use the same approach for all equations in order to estimate which equation is best for CHF patients regardless of their kidney function. The influence of body composition on the accuracy of GFR estimation was studied in both sexes, not taking into consideration the body composition differences between the sexes. Renal function declined during follow-up, but the same protocol for renal function measurement with iohexol was applied at both visits. For patients with GFR<30 mL/(min ×1.73 m^2^), this could be relevant as iohexol clearance is expected to overestimate renal function with sampling times shorter than 4 hours. However, this was clearly demonstrated only for one-sample protocol (33), while we determined iohexol clearance at two time points. The patients in whom mGFR increased during follow up were found in all ranges of mGFR, so the increase in mGFR is not likely to be due to inadequate sampling time. Moreover, if the overestimation was indeed present, our results would be even more alarming as true renal function would be even lower than actually measured.

In conclusion, mGFR outperformed the equations in CHF patients, and did so across all stages of chronic kidney disease. Patients receiving mineralocorticoid receptor antagonists were more prone to develop a rapid decline in renal function. All tested equations correlated well with mGFR, but their overestimation increased as mGFR decreased. Due to their dependence on body composition parameters, CG and CKD-EPI equations should be used with caution in extremely underweight or obese patients. In clinical practice, eGFR appears to be insufficient to detect clinically relevant changes in renal function, which is why pharmacological therapy may be delayed or not modified, exposing some of the patients to increased risk of complications. However, eGFR cannot be replaced by an exogenous marker of renal function, but rather with a different endogenous marker. Our results also imply that eGFR may not be an ideal primary end-point in patients with CHF. Further studies with larger sample size and longer follow-up are needed to clarify this issue.

## References

[1] Khan NA, Ma I, Thompson CR, Humphries K, Salem DN, Sarnak MJ (2006). Kidney function and mortality among patients with left ventricular systolic dysfunction.. J Am Soc Nephrol.

[2] Damman K, Valente MAE, Voors AA, O’Connor CM, van Veldhuisen DJ, Hillege HL (2014). Renal impairment, worsening renal function, and outcome in patients with heart failure: an updated meta-analysis.. Eur Heart J.

[3] Stevens LA, Levey AS (2009). Measured GFR as a confirmatory test for estimated GFR.. J Am Soc Nephrol.

[4] Brown SC, O’Reilly PH (1991). Iohexol clearance for the determination of glomerular filtration rate in clinical practice: evidence for a new gold standard.. J Urol.

[5] Gaspari F, Perico N, Ruggenenti P, Mosconi L, Amuchastegui CS, Guerini E (1995). Plasma clearance of nonradioactive iohexol as a measure of glomerular filtration rate.. J Am Soc Nephrol.

[6] Cockcroft DW, Gault MH (1976). Prediction of creatinine clearance from serum creatinine.. Nephron.

[7] Levey AS, Bosch JP, Lewis JB, Greene T, Rogers N, Roth D (1999). A more accurate method to estimate glomerular filtration rate from serum creatinine: a new prediction equation. Modification of Diet in Renal Disease Study Group.. Ann Intern Med.

[8] Levey AS, Stevens LA, Schmid CH, Zhang YL, Castro AF, Feldman HI (2009). A new equation to estimate glomerular filtration rate.. Ann Intern Med.

[9] Valente MA, Hillege HL, Navis G, Voors AA, Dunselman PH, van Veldhuisen DJ (2014). The Chronic Kidney Disease Epidemiology Collaboration equation outperforms the Modification of Diet in Renal Disease equation for estimating glomerular filtration rate in chronic systolic heart failure.. Eur J Heart Fail.

[10] Plischke M, Neuhold S, Kohl M, Heinze G, Sunder-Plassmann G, Pacher R (2013). Renal function in heart failure: a disparity between estimating function and predicting mortality risk.. Eur J Heart Fail.

[11] McAlister FA, Ezekowitz J, Tarantini L, Squire I, Komajda M, Bayes-Genis A (2012). Renal dysfunction in patients with heart failure with preserved versus reduced ejection fraction: impact of the new Chronic Kidney Disease-Epidemiology Collaboration Group formula.. Circ Heart Fail.

[12] Patel SS, Molnar MZ, Tayek JA, Ix JH, Noori N, Benner D (2013). Serum creatinine as a marker of muscle mass in chronic kidney disease: results of a cross-sectional study and review of literature.. J Cachexia Sarcopenia Muscle..

[13] Macdonald JH, Marcora SM, Kumwenda MJ, Jibani M, Roberts G, Glover R (2006). The relationship between estimated glomerular filtration rate, demographic and anthropometric variables is mediated by muscle mass in non-diabetic patients with chronic kidney disease.. Nephrol Dial Transplant.

[14] Ozmen S, Kaplan MA, Kaya H, Akin D, Danis R, Kizilkan B (2009). Role of lean body mass for estimation of glomerular filtration rate in patients with chronic kidney disease with various body mass indices.. Scand J Urol Nephrol.

[15] Hillege HL, Nitsch D, Pfeffer MA, Swedberg K, McMurray JJ, Yusuf S (2006). Renal function as a predictor of outcome in a broad spectrum of patients with heart failure.. Circulation.

[16] Smith GL, Lichtman JH, Bracken MB, Shlipak MG, Phillips CO, DiCapua P (2006). Renal impairment and outcomes in heart failure: systematic review and meta-analysis.. J Am Coll Cardiol.

[17] Xie D, Joffe MM, Brunelli SM, Beck G, Chertow GM, Fink JC (2008). A comparison of change in measured and estimated glomerular filtration rate in patients with nondiabetic kidney disease.. Clin J Am Soc Nephrol.

[18] Padala S, Tighiouart H, Inker LA, Contreras G, Beck GJ, Lewis J (2012). Accuracy of a GFR estimating equation over time in people with a wide range of kidney function.. Am J Kidney Dis.

[19] Nilsson-Ehle P. Iohexol clearance for the determination of glomerular filtration rate: 15 years ´ experience in clinical practice. eJIFCC. 2002;13(2). Available from: http://www.ifcc.org/ejifcc/vol13no2/1301200105.htm. Accessed: September 16, 2015. PMC6232857 30429722

[20] Bröchner-Mortensen J (1972). A Simple method for the determination of glomerular filtration rate.. Scand J Clin Lab Invest.

[21] Du Bois D, Du Bois EF (1989). A formula to estimate the approximate surface area if height and weight be known. 1916.. Nutrition.

[22] Janssen I (2004). Skeletal muscle cutpoints associated with elevated physical disability risk in older men and women.. Am J Epidemiol.

[23] Soman RS, Zahir H, Akhlaghi F (2005). Development and validation of an HPLC-UV method for determination of iohexol in human plasma.. J Chromatogr B Analyt Technol Biomed Life Sci.

[24] de Silva R, Nikitin NP, Witte KK, Rigby AS, Goode K, Bhandari S (2006). Incidence of renal dysfunction over 6 months in patients with chronic heart failure due to left ventricular systolic dysfunction: contributing factors and relationship to prognosis.. Eur Heart J.

[25] Juurlink DN, Mamdani MM, Lee DS, Kopp A, Austin PC, Laupacis A (2004). Rates of hyperkalemia after publication of the Randomized Aldactone Evaluation Study.. N Engl J Med.

[26] Maeder MT, Rickli H, Pfisterer ME, Muzzarelli S, Ammann P, Fehr T (2012). Incidence, clinical predictors, and prognostic impact of worsening renal function in elderly patients with chronic heart failure on intensive medical therapy.. Am Heart J.

[27] Shlipak MG (2003). Pharmacotherapy for heart failure in patients with renal insufficiency.. Ann Intern Med.

[28] O’Meara E, Chong KS, Gardner RS, Jardine AG, Neilly JB, McDonagh TA (2006). The Modification of Diet in Renal Disease (MDRD) equations provide valid estimations of glomerular filtration rates in patients with advanced heart failure.. Eur J Heart Fail.

[29] Smilde TDJ, van Veldhuisen DJ, Navis G, Voors AA, Hillege HL (2006). Drawbacks and prognostic value of formulas estimating renal function in patients with chronic heart failure and systolic dysfunction.. Circulation.

[30] Ruggenenti P, Gaspari F, Cannata A, Carrara F, Cella C, Ferrari S (2012). Measuring and estimating GFR and treatment effect in ADPKD patients: results and implications of a longitudinal cohort study.. PLoS ONE.

[31] Gaspari F, Ruggenenti P, Porrini E, Motterlini N, Cannata A, Carrara F (2013). The GFR and GFR decline cannot be accurately estimated in type 2 diabetics.. Kidney Int.

[32] Thomas MC, Jerums G, Tsalamandris C, Macisaac R, Panagiotopoulos S, Cooper ME (2005). Increased tubular organic ion clearance following chronic ACE inhibition in patients with type 1 diabetes.. Kidney Int.

[33] Sterner G, Frennby B, Hultberg B, Almen T (1996). Iohexol clearance for GFR-determination in renal failure–single or multiple plasma sampling?. Nephrol Dial Transplant.

