# Supplementary material for: Iohexol clearance is superior to creatinine-based renal function estimating equations in detecting short-term renal function decline in chronic heart failure
Source: Croat Med J. 2015 Dec;56(6):531–41. doi: 10.3325/cmj.2015.56.531 (PMC4709563; doi:10.3325/cmj.2015.56.531)

Supplementary Figure 1. Decline in eGFR during follow up (N=31)

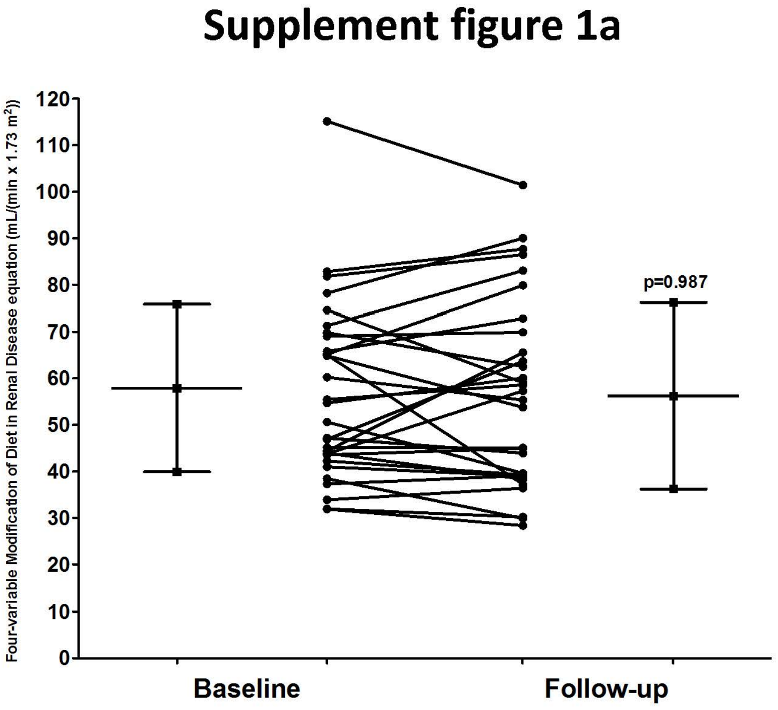

Supplement figure 1b

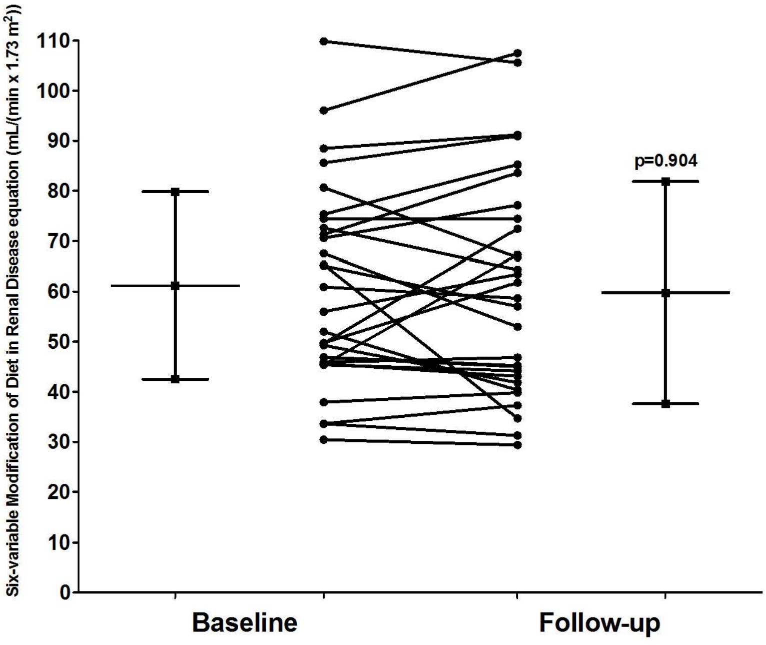

Supplement figure 1c

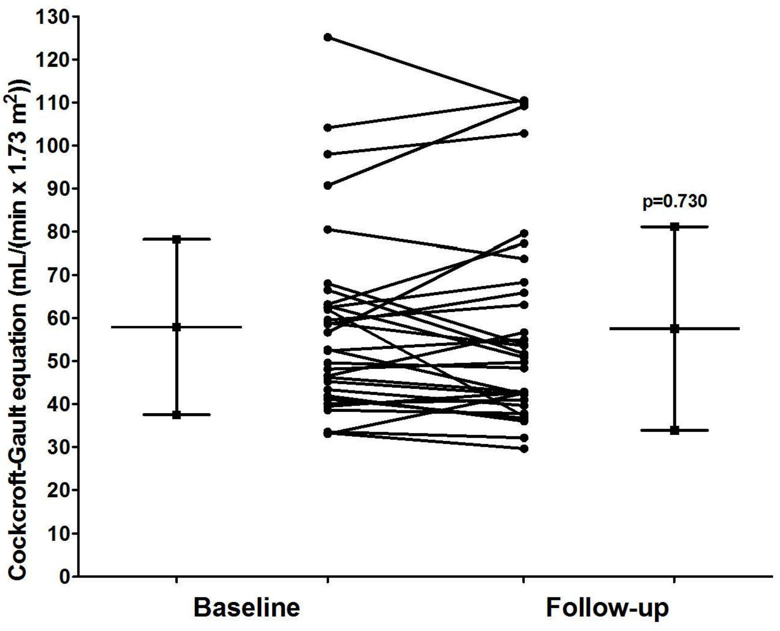

Supplement figure 1d

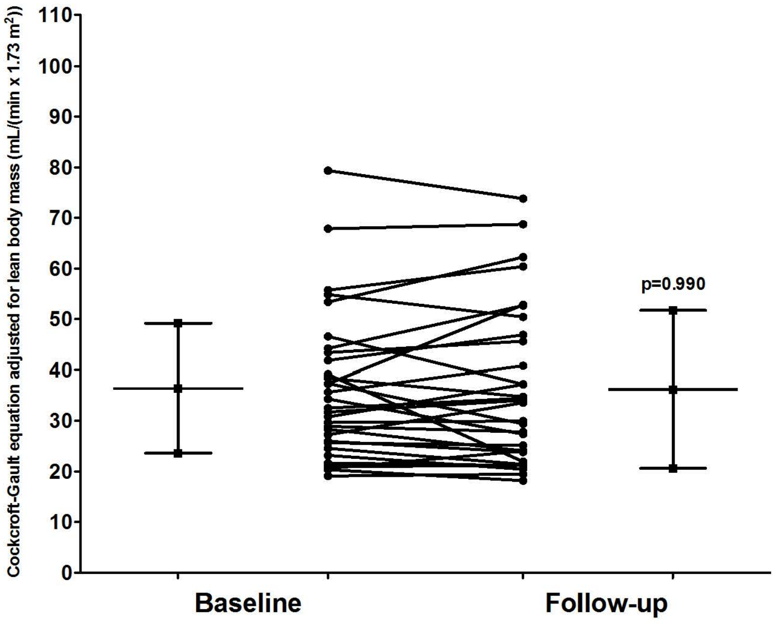

Supplement figure 1e

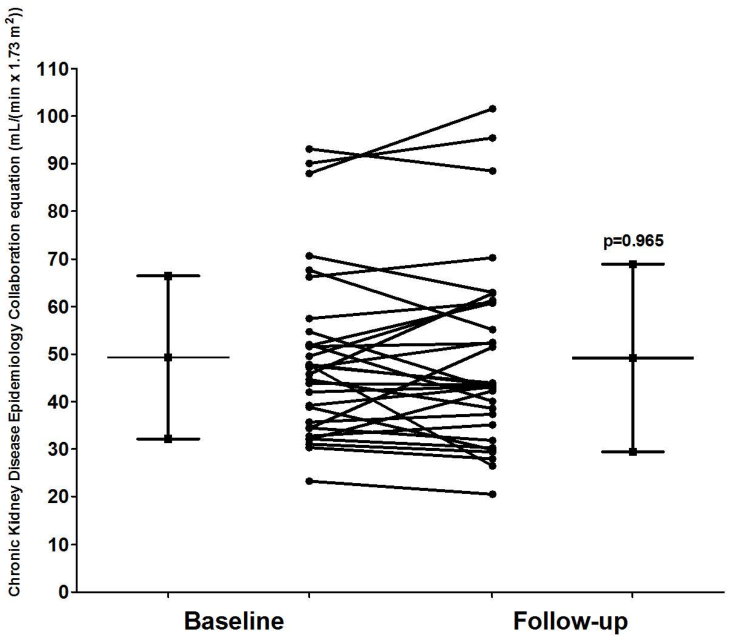

Supplement: Supplementary Figure 1 [file CroatMedJ_56_s001.pdf]
