# Supplementary material for: Iohexol clearance is superior to creatinine-based renal function estimating equations in detecting short-term renal function decline in chronic heart failure
Source: Croat Med J. 2015 Dec;56(6):531–41. doi: 10.3325/cmj.2015.56.531 (PMC4709563; doi:10.3325/cmj.2015.56.531)
Supplement: Supplementary Table 1 [file CroatMedJ_56_s002.pdf]

Supplementary table 1: Correlation of percentage errors and body composition parameters at baseline (Pearson coefficient, p-value)

	PE MDRD4	PE MDRD6	PE CG	PE CGLBM	CKD-EPI
Body weight (kg)	NS	NS	**0.573 (<0.001)**	0.353 (0.020)	NS
BSA	NS	NS	0.415 (0.006)	0.310 (0.043)	-0.383 (0.011)
BMI	NS	NS	**0.701 (<0.001)**	0.346 (0.023)	NS
SMI	NS	NS	NS	0.418 (0.006)	-0.348 (0.024)
FFMI	NS	NS	0.356 (0.021)	0.386 (0.012)	-0.344 (0.026)
**BIA (N= 39)**					
Fat (kg)	NS	NS	**0.617 (<0.001)**	NS	NS
Fat (%)	NS	NS	0.348 (0.030)	NS	**0.602 (<0.001)**
Lean (kg)	NS	NS	NS	0.414 (0.009)	**-0.532 (<0.001)**
Lean (%)	NS	NS	-0.335 (0.037)	NS	**-0.605 (<0.001)**
Dry lean (kg)	NS	NS	0.336 (0.036)	0.441 (0.005)	**-0.502 (0.001)**
Dry lean (%)	NS	NS	NS	0.378 (0.018)	**-0.571 (<0.001)**
Water (L)	NS	NS	NS	0.375 (0.019)	**-0.545 (<0.001)**
Water (%)	NS	NS	**-0.620 (<0.001)**	NS	-0.388 (0.015)
**DEXA (N= 42)**					
Fat (kg)	NS	NS	**0.744 (<0.001)**	NS	NS
Fat (%)	NS	NS	**0.724 (<0.001)**	NS	**0.545 (<0.001)**
Lean (kg)	NS	NS	NS	NS	**-0.579 (<0.001)**
Lean (%)	NS	NS	**-0.727 (<0.001)**	NS	**-0.538 (<0.001)**

BSA = body surface area, BMI = body mass index, SMI = skeletal muscle index, FFMI = fat-free mass index, BIA = bioimpedance, DEXA = dual-energy X-ray absorptiometry, PE = percentage error, MDRD4 = four-variable Modification of Diet in Renal Disease equation, MDRD6 = six-variable Modification of Diet in Renal Disease, CG = Cockcroft-Gault equation, CGLBM =Cockcroft-Gault equation adjusted for lean body mass, CKD-EPI= Chronic Kidney Disease Epidemiology Collaboration equation, NS = not significant at the level of 0.05
