# Supplementary material for: Iohexol clearance is superior to creatinine-based renal function estimating equations in detecting short-term renal function decline in chronic heart failure
Source: Croat Med J. 2015 Dec;56(6):531–41. doi: 10.3325/cmj.2015.56.531 (PMC4709563; doi:10.3325/cmj.2015.56.531)
Supplement: Supplementary Table 2 [file CroatMedJ_56_s003.pdf]

Supplementary table 2: Hierarchical multiple linear regression analysis explaining variance in measured glomerular filtration rate. All models were statistically significant (p< 0.001).

	R^2	R^2_{change}	F_{change}	Sig. F_{change}	β	B	Intercept
MDRD4	0.708	0.708	96.878	0.000	0.795	0.777	-14.220
% lean	0.735	0.027	3.934	0.054	0.170	0.348	
MDRD6	0.693	0.693	87.895	0.000	0.792	0.744	-16.162
% lean	0.721	0.029	3.910	0.055	0.174	0.374	
CG	0.525	0.025	44.238	0.000	0.784	0.685	-50.404
% lean	0.756	0.230	36.745	0.000	0.484	0.989	
CGLBM	0.719	0.719	102.179	0.000	0.801	1.112	-14.411
% lean	0.758	0.040	6.387	0.016	0.204	41.817	
CKD-EPI	0.524	0.524	44.044	0.000	0.726	0.739	-34.779
% lean	0.677	0.153	18.498	0.000	0.391	80.050	

R^2 = accumulative explained variance in measured glomerular filtration rate, β = standardized coefficient, B = unstandardized coefficient, MDRD4 = four-variable Modification of Diet in Renal Disease equation, MDRD6 = six-variable Modification of Diet in Renal Disease equation, CG = Cockcroft-Gault equation, CGLBM =Cockcroft-Gault equation adjusted for lean body mass, CKD-EPI = Chronic Kidney Disease Epidemiology Collaboration equation
